# Supplementary material for: Signatures of Long-Term Balancing Selection in Human Genomes
Source: Genome Biol Evol. 2018 Mar 15;10(3):939–55. doi: 10.1093/gbe/evy054 (PMC5952967; doi:10.1093/gbe/evy054)
Supplement: Supplementary Materials [file evy054_supp.zip › Supplementary_Material_revised.docx]

**Supplementary Material Online**

Signatures of long-term balancing selection in human genomes

Bárbara Domingues Bitarello1,2,*, Cesare de Filippo2, João Carlos Teixeira2,3, Joshua M. Schmidt2, Philip Kleinert2,4, Diogo Meyer1, ¶ ,*, Aida M. Andrés2, 5,¶,*

1, Department of Genetics and Evolutionary Biology, University of São Paulo, São Paulo, Brazil

2, Evolutionary Genetics Department, Max Planck Institute for Evolutionary Anthropology, Leipzig, Germany

3, Unit of Human Evolutionary Genetics, Institut Pasteur, Paris, France

4, Current address: Computational Molecular Biology Department, Max Planck Institute for Molecular Genetics, Berlin, Germany

5, UCL Genetics Institute, Department of Genetics, Evolution and Environment, University College London, London

¶, AA and DM co-supervised the study

*Corresponding author(s): [barbara_domingues@eva.mpg.de](mailto:barbara_domingues@eva.mpg.de) (BDB),  [diogo@ib.usp.br (DM)](mailto:diogo@ib.usp.br)), [aida_andres@eva.mpg.de](mailto:aida_andres@eva.mpg.de) (AMA).

**Supplementary Information S1*.* NCD method and power analyses**

*Frequency equilibrium*

With overdominance, given selection coefficients *s* and *t* against the *AA* and *BB* homozygotes*,* respectively, the deterministic frequency equilibrium (*f*eq) is given by:

and (1)

Under symmetric overdominance, selection coefficients of both homozygotes are the same (), and thus *f*eq = 0.5. With asymmetric overdominance, homozygotes have different fitnesses (*t* ≠ *s*) - which might be more prevalent in natural systems (Hedrick 2012) - and it follows that *f*eq ≠ 0.5.

A classic example of asymmetric overdominance is the case of the *β*-globin mutation (*HbS*), which causes sickle cell anemia. In regions of endemic malaria the fitness of the *HbA/HbS* heterozygotes is the highest, while the *HbA* homozygote has fitness approximately nine times higher than that of the *HbS* homozygote, with the resulting equilibrium frequency of the *Hbs* allele being 0.13 (Allison and Clyde 1961).

Under frequency-dependent selection, *f*eq will depend on the frequency of the favored allele, and under fluctuating selection, the selected allele’s frequency will depend on the temporal and spatial scales of selection (Charlesworth and Charlesworth 2010).

*NCD properties*

The lower the NCD value, the lower the average distance between the SNP frequencies and the *tf*. From equation 1 (main paper) it follows that the maximum value for *NCD2(tf)* is the *tf* itself (for *tf* ≥ 0.25), which occurs when there are no SNPs and the number of FDs ≥ 1.The maximum *NCD1*value approaches – but never reaches – *tf* when all SNPs are singletons. The minimum possible value for both *NCD1*and *NCD2*is 0, when all SNPs segregate at tf and, in the case of *NCD2*, the number of FDs = 0 (figs. S1 and S2). Because *NCD* equations use the folded SFS, the highest allele frequencies are 0.5. This means that, if *tf* is greater than or equal to 0.25 (which is half of the maximum allele frequency possible), the maximum distance to *tf* is *tf* itself. When *tf* is lower than 0.25, say 0.2, then the maximum *NCD* value would be found when allele frequencies are 0.5, in which case the distance would be 0.5-0.2=0.3, i.e, higher than the *tf.* For *tf* < 0.5/2, the maximum *NCD* value is 0.5-tf (e.g., if *tf* = 0.2, the maximum *NCD* is 0.3, i.e, 0.5-0.2). In such cases, the interpretation of *NCD* values is less straightforward since low values are also compatible with neutrality and purifying selection. We thus explore only *tf* = {0.3, 0.4, 0.5}.

*Simulating balancing selection*

Under the overdominance model, for a bi-allelic locus with alelles A and B, the relative fitnesses of the three genotypes are: wAA = 1 – *s*, wAB = 1, and wBB = 1 – *t*, where *s* and *t* are the selection coefficients of the two homozygous genotypes, and the frequency equilibrium (*f*eq) is equal to *s*/(*s*+*t*), as in Equation 1.

We simulated sequences evolving neutrally and under balancing selection using MSMS (Ewing and Hermisson 2010). In order to achieve the desired *f*eq, we parameterized selection in the following way: , , and, where *N*e is the effective population size used to scale the coalescent simulations and *s* is the selection coefficient for one of the alleles. The selection coefficient is always set to *s* = 0.01, since the distance from the *tf* is determined by the proportional difference in selection against homozygotes, not the *s* value itself. For simulations with selection, we only retained those where the balanced polymorphism is still segregating at the time of sampling.

*Power analyses*

***Implementation of NCD.*** Power for *NCD2* is greater than for *NCD1* for all *tf*: *f*eq = 0.5 (average power of 0.94 for *NCD2(0.5)* vs. 0.88 for *NCD1(0.5),* averaged across populations and *Tbs*; table 1)*,* *f*eq = 0.4 (0.90 for *NCD2(0.4)* vs. 0.80 for *NCD1(0.4)*) and *f*eq = 0.3 (0.86 for *NCD2(0.3)* vs. 0.73 for *NCD1(0.3)*) (table 1, Figure 2). The main properties discussed for *NCD2* in the main paper are also true for *NCD1*: higher power for smaller windows, and for ancient selection (see below). On the other hand, sample size impacts *NCD1* more than *NCD2* (see below and table S1).

When *NCD1* is combined with HKA, we see an increment in power compared to *NCD1* alone (S1 table) which further demonstrates that by adding information from fixed differences there is gain in power (which is why *NCD2* outperforms *NCD1*).

**Sequence length and *Tbs***. *NCD* power is higher for 3kb regions than for 12kb regions.For *NCD1,* the power increment for shorter regions is less pronounced than for *NCD2* (table S1),likely due to the lower number of informative sites increasing noise (*NCD1* only uses SNPs, whereas *NCD2* uses SNPs and FDs). This is true not only for *NCD(0.5)* (about 10% reduction in powerfor 12 kb compared to 3 kb*)*, but a similar picture emerges for *NCD(0.4)*(with 19% reduction in power) and *NCD(0.3)* (24% reduction in power) (table S1; figs. S3-S8). When comparing *NCD1* and *NCD2*, we note that the power increment for 3 kb compared to 6 kb is stronger for *NCD2* than for *NCD1*; in the latter, in a few instances power for 6 kb may be slightly larger for 6 kb. This is likely due to the fact that narrow windows have a stronger effect on the number of available informative sites (IS) for *NCD1* than for *NCD2* (SNPs + FDs). Regardless, the overall trend is that power is similar between 3 and 6 kb, and in most cases higher for 3 kb. For *NCD1*, the power trend regarding *Tbs* is similar to *NCD2*: 5 > 3 > 1 mya.

**Frequency equilibrium (*f*eq) of** **0.2.**Selection with *f*eq = 0.2 results in low power across all parameters and *tf* values (figs. S3-S8), so we do not further explore this target frequency.

**Demographic scenarios.**The power under the Asian scenario is lower than that of the African and European scenarios (average *NCD(0.5)* of 0.48). One possible explanation is the lower *Ne* (e.g.(Gutenkunst et al. 2009; Gravel et al. 2011)): lower *Ne* causes random genetic drift to be stronger in this population, affecting the SFS under both neutrality and balancing selection. In neutral loci, lower *Ne* can result in a higher proportion of alleles at intermediate frequency, which makes the neutral SFS more similar to the expected SFS under LTBS. This is observed when comparing the SFS of Asian populations to those of African and European ones (Gutenkunst et al. 2009). In loci under LTBS, lower *Ne* implies in reduced efficacy of balancing selection, and could result in the selected alleles being less efficiently maintained close to the *feq*, making the SFS more similar to that expected under neutrality. Both effects would reduce the power of NCD.

Also, unlike *NCD2*, for which African and European simulations have very similar power values in all cases, for *NCD1* we see that power is similar for these populations in most cases, but is considerable lower for Europe when *tf* = 0.3 and *f*eq = 0.5 (table S1), while still high in both when *tf* = *f*eq = 0.3, which speaks in favor of considering different target frequencies when scanning the genome.

**Sample size.** Power analyses reported the main text were derived from sampling 100 chromosomes (50 diploid individuals) for each population. We additionally explored power for samples of 60 and 20 chromosomes (table S1).

For *NCD2*, the most trends are the same across sample sizes, in that: power is highest for 3 kb and lowest for 12 kb; highest for 5 mya and lowest for 1 mya; similar for African and European simulations, but considerably lower for Asian simulations; *NCD2* has higher power than *NCD1*. For the small sample size (n=20), the trend of power 3 kb > 6 > 12 kb does not always hold for *Tbs* = 1 mya and in some instances of *Tbs* = 3 mya, where it may occur that 6 > 3 > 12 kb. For the Asian simulations, at n = 20 and *Tbs* = 1 mya, we see cases where power is higher for 12 than for 3 kb, even though the average power for any length given those conditions is extremely low. These patterns likely result from the reduced number of SNPs segregating in the simulations with a low sample size and low *Tbs*, which is partially compensated by an increase in window size. In Asia, the even more aberrant patterns may be due to the effect of the lower *N*e on the number of segregating sites.

More importantly, in the timescale and window length at which *NCD2* performs best (*Tbs* = 3, 5; *L* = 3 kb), both n=100 and n=60 yield similar power values, and those are consistently higher than for n = 20.

For *NCD1*, the main trends also hold regardless of sample size: as with n = 100, power tends to be highest for either 3 or 6 kb; highest for *Tbs* = 5 and lowest for *Tbs* = 1 mya; power is similar for African and European simulations, except when *feq* = 0.5 and *tf* = 0.3, when Africa has higher power (this is true for sample sizes of 50 and 30 individuals). On the other hand, there is a clear pattern of overall power reduction when n = 20, unlike *NCD2*, where power values remain high even for small sample size. This is likely due to the fact that the reduction in polymorphic sites caused by small sample sizes impacts *NCD1* more strongly than *NCD2* because those are the only informative sites considered by *NCD1*, whereas *NCD2* also takes FDs into account.

**Other neutrality tests.** We simulated 15 kb windows and calculated *T*1 and *T*2 with BALLET (DeGiorgio et al. 2014) for windows of 100 IS for simulations with African and European demography, and selected the highest *T*1 or *T*2 value from each simulation to obtain their power for the same set of parameters used for the other simulations. We again used 15 kb windows from simulations for African and European demographic scenarios to calculate ß for windows of 1, 2 and 3 kb. The highest value from each simulation (neutral or with selection) was chosen, and power was verified at FPR=5%. When focusing on the tests that use only polymorphic sites, *NCD1* has similar power to Taj*D* when *f*eq = 0.5, and it outperforms it when *f*eq departs from 0.5 (table S1). Power for *T*1 and *T*2 is extremely similar to those originally reported by DeGiorgio et al. (2014), and our power for Taj*D* is substantially higher than inDeGiorgio et al. (2014) (probably due to the choice of window size and different models).

**Supplementary Information S2*.* Genome-wide scan**

*Filtering out windows with few informative sites*

Genome-wide studies of natural selection typically place a threshold on the minimum number of ISs in the queried window (e.g. at least 10 in (Andrés et al. 2009), or 100 in (DeGiorgio et al. 2014). We observe considerable variance in the number of ISs per 3 kb window in the 1000G data and find that *NCD2* has high variance when the number of ISs is low in neutral simulations (figs. S11 and S17 and information S2). We therefore excluded windows with less than 10 ISs in a given population, and retained the remainder since above that threshold *NCD2* stabilizes (fig. S11).

*Correcting NCD2 values for number of informative sites*

Neutral simulations with different mutation rates were performed in order to retrieve 10,000 simulations for each value of IS observed in the real data (fig. S17). *NCD*2 (with *tf* = 0.3, 0.4, 0.5) was calculated for all simulations, allowing the assignment of scanned windows and the calculation of *Ztf-IS*(Eqn. 2, main).

*Assigned tf values*

When a window is an outlier for several *tf* values, we sought to identify the *tf* value that minimizes *NCD2(tf).* As described in the main text (section “Assigned *tf* values”), the *p*-values obtained from *Ztf-IS*were used to identify the *tf* value that yields the lowest empirical *p*-value for the window (tables S3 and S4).

Among the candidate windows for the three *tf* values, on average 52% and 53% (outlier and significant windows, respectively) are assigned to *tf* = 0.3 (table S3). For the outlier windows, the proportion of assigned windows to 0.4 is 31% and to 0.5 is 17% (almost 3-fold difference between 0.3 and 0.5). For the significant windows, 0.4 (22%) and 0.5 (25%) have similar proportions of windows assigned to them.

For the outlier genes in table S4, the assigned *tf* value for a gene is for the window with the lowest empirical *p*-value, and was defined for each population separately. Most genes have the same assigned *tf* value in different populations: 67% of the African genes have the same assigned *tf* in LWK and YRI, and 63 % of the European genes have the same assigned *tf* for GBR and TSI. Finally, 86% of the shared genes have the same assigned *tf* for *at least* two populations from the same continent and often (46%) for all populations.

*Coverage as a proxy for undetected short duplication*

To test whether our signatures of LTBS are driven by undetected short duplications, which can produce mapping and SNP call errors, we analyzed an alternative human genome-wide dataset, sequenced to an average coverage of 20x-30x per individual (Meyer et al. 2012; Prüfer et al. 2013). We used an independent dataset because read coverage data is low and cryptic in the 1000G, and putative duplications affecting the SFS must be at appreciable frequency and should be present in other data sets. We considered 2 genomes from each of the following populations: Yoruba, San, French, Sardinian, Dai, and Han Chinese. For each sample, we retrieved positions above the 97.5% quantile of the coverage distribution for that sample (“high coverage” positions). For each window with signatures of LTBS, we calculated the proportion of the 3kb window having high coverage in at least two samples and plotted the distributions for different *NCD*2 *Ztf-IS* *p*-values.

**Supplementary Information S3. Ruling out possible biological confounding factors**

We explored the influence of two possible biological confounding factors, which could increase genetic diversity in the absence of balancing selection: Neandertal introgression (only for European candidates) and ectopic gene conversion. Here, the set of significant or outlier windows is defined as the union of windows, considering all *tf* (0.3, 0.4, 0.5)values. The genes are those overlapped by those windows.

*Neandertal introgression*

**Background.** Genomic regions that contain introgressed haplotypes from archaic hominins (Denisova, Neandertal)have, on average, slightly older time to the most recent ancestor and higher diversity than the rest of the genome. This provides some potential for finding candidates of LTBS in Europeans – and absent in Africans – that are rather due to introgression. However, in the absence of balancing (or positive) selection, introgressed segments are not expected to reach intermediate frequencies (minor allele frequency, MAF ≥ 0.3). As *NCD2* requires both intermediate frequencies of SNPs and greatly proportion of FDs to SNPs, we do not expect that introgressed regions would contribute greatly to the candidate windows defined in the main paper. We provide the following results to show that this is indeed true.

**The proportion of introgressed SNPs**. We independently tested the enrichment of Neandertal introgression among candidate windows in TSI and GBR by using the re-sampling approach described for functional enrichment analysis (information S3, below). Putative Neandertal-introgressed SNPs were ascertained per population as follows: 1) select SNPs that pass our filters and that are in the candidate windows; 2) select the SNPs that segregate in each of the European populations while being monomorphic in YRI; 3) select the SNPs that also overlap a segment inferred to be of Neandertal origin; 4) select the SNPs that match one of the alleles carried by the Altai Neandertal (Prüfer et al. 2013), and where Altai has at least one allele different to all YRI. The proportion of all SNPs that are putatively Neandertal-introgressed was computed independently for GBR and TSI following the steps above, for each window set (outlier and significant, all *tf* values) and for three maps of Neandertal introgression (Sankararaman et al. 2014; Vernot and Akey 2014; Vernot et al. 2016). Where provided (Sankararaman et al. 2014; Vernot and Akey 2014) we used matching population specific haplotype calls, and merged haplotypes across individuals; in the case of the map from (Vernot et al. 2016) we used the combined European haplotypes. Observed values were compared to those obtained by 1,000 re-samplings of the same number of windows from all the scanned windows.

For both GBR and TSI we find that significant and outlier windows are not enriched in putatively Neandertal introgressed SNPs (defined in methods below), and are in fact significantly depleted of them (fig. S16). This is consistent between the three introgression maps, referred to as v2014 (Vernot and Akey 2014), v2016 (Vernot et al. 2016) and s2014 (Sankararaman et al. 2014). In keeping with expectations given the average fraction of a European genome that is of Neandertal ancestry, we estimate the background proportion introgressed SNPs as 1-1.8 %. In contrast, significant and outlier windows range from 0.4-0.8% (reduction in introgressed SNPs ~ 50%).

**Individual haplotypes**. Of course, finding a depletion of introgressed SNPs across the set of candidate windows does not preclude that specific windows are *NCD2* outliers due to introgression. We designated as putatively introgressed SNPs if 1) they fall in a window that is not present in the union of the equivalent YRI and LWK sets and is present in any map of Neandertal introgression; 2) the proportion of SNPs in the window that are introgressed is ≥ 0.25; 3) the median MAF of introgressed SNPs in the window is ≥ 0.2.

We find that for GBR and TSI there are only four and five outlier windows (four in both populations) and 22 and 20 significant windows (15 in both populations), respectively, that match our criteria as an intermediate frequency Neandertal haplotype (table S4).

***Ectopic gene conversion***

We also investigated the possibility of ectopic gene conversion, which is another biological phenomenon that may increase diversity. This is because paralogs that are close to each other on a chromosome may incorporate segments from one another through ectopic gene conversion (also known as paralogous gene conversion), increasing diversity even in the absence of balancing selection.

For each gene identified as significant in LWK (see main text), we analyzed the distribution of the number of paralogs that reside on the same chromosome. The set of significant genes does not show a systematic trend towards having more paralogs on the same chromosome than other autosomal genes (seefig.S15), showing that this is not a general issue. In both cases, more than 60% of the genes have no paralogs on the same chromosome.

We nevertheless singled out olfactory receptor (OR) genes, which often appear in tandem and may undergo ectopic gene conversion. Unlike the other significant and background genes, more than 80% of the significant OR genes in LWK have at least one paralog on the same chromosome (fig. S15). Thus, ectopic gene conversion does not appear to be a general issue among significant genes, with the exception of the OR genes.

**Supplementary Information S4. Functional enrichment analyses of candidate windows and their SNPs**

*Gene Ontology of windows*

We constructed gene annotation (.gtf) and gene set (GO accession terms and associated genes) files to run GOWINDA. In order to build the .gtf file, we started by downloading from ENSEMBL biomart the hg19 chromosome, start and end coordinates, ENSEMBL gene identifiers and external gene names for each autosomal, known, protein coding gene (18,564 genes total; accessed February, 2016). 26 genes in ENSEMBL have at least two ENSEMBL gene IDs associated with the same external gene name, which can lead to the same gene being associated with the same GO term more than once, and/or different annotations of the gene being associated with different GO terms. This can have an adverse effect on the sampling estimates of GO enrichment, particularly if these “duplicates” are overlapping in the genome. We solved these inconsistencies in an iterative fashion. 1) We renamed genes with the same name but located on different chromosomes by affixing the chromosome number. In hg19 there is only one gene, *CKS1B*, located on chromosomes 1 and 5, with this problem. (NB. for Grch38, *CKS1B* on chromosome 5 is now named *CKS1BP3*, and is annotated as a pseudogene.) 2) If the different coordinates of the gene were located in the same chromosome but overlapping, we merged all gene coordinates to create a single gene entry, taking the minimum start and maximum end positions as the new gene start and end positions, respectively. 3) For disjoint duplicates, we simply renamed geneX as geneX_a, geneX_b, geneX_c etc. as required. This happens only for a few genes. The .gtf file was then compiled from this modified gene set.

We downloaded the go-basic.obo and goa_human.gaf files from the Gene Ontology Consortium (accessed Februrary, 2016). A custom *perl* script was used to parse the go-basic.obo file to output a table of GO terms and their definitions. Gene names (matching ensemble external gene names mentioned above) and associated GO terms were extracted from the goa_human.gaf file, making sure that the associated protein taxon was human (i.e. 9606). For the duplicate coordinate genes identified above, we gave each gene copy the same GO term associations as for their parent gene.

GOWINDA was designed for SNP-based analysis so we considered the middle position of every scanned window as the target site and extended gene coordinates by 1,500 bp up/down-stream by using the option *updownstream1500* in SNP to gene mapping (to analyze the full 3kb region).

The gene enrichment results are presented in the main text (section “Biological pathways influenced by LTBS”) and in tables S2-S4. In some analyses, the following set of HLA genes was excluded to explore the influence of balancing selection outside of the MHC locus, because these have prior evidence of LTBS*: HLA-B*, *HLA-C*, *HLA-DRB1*, *HLA-DRB5*, *HLA-DPA1*, *HLA-DPA2*, *HLA-DPB1*, *HLA-DPB2*, *HLA-DQB1*, *HLA-DQB2*, *HLA-DQA1*, *HLA-DQA2*(Tan et al. 2005; Liu et al. 2006; Meyer et al. 2006; Sanchez-Mazas 2007; Solberg et al. 2008; DeGiorgio et al. 2014; Teixeira et al. 2015)***.***

*Annotated function of SNPs*

We also used ENSEMBL to determine the functional annotation of individual SNPs. We defined as 1) intergenic SNPs those annotated as: “*transcript ablation”, “transcript amplification”, “mature miRNA variant”, “NMD transcript variant”, “non-coding transcript variant”, “upstream gene variant”, “downstream gene variant”, “TFBS ablation”, “TFBS amplification”, “TF binding site variant”, “regulatory region ablation”, “regulatory region amplification”, “feature elongation,” “regulatory region variant”, “feature truncation”, “intergenic variant”*;2) genic SNPs all the ones not annotated as intergenic (as above); 3) synonymous SNPs those annotated as *synonymous*; 4) non-synonymous SNPs those annotated as *missense*; 5) exonic SNPs those annotated as “*stop gained”, “frameshift variant”, “stop lost”, “start lost”, “stop gained”, “inframe insertion”, “inframe deletion”, “missense variant”, “protein altering variant”, “incomplete terminal codon variant”, “stop retained variant”, “synonymous variant”*; 6) regulatory SNPs those annotated as “*TFBS ablation*”*,*“*TFBS amplification*”*,*“*TF binding site variant*”*,* “*regulatory region ablation*”*,* “*regulatory region amplification*”*,* “*feature elongation*”*,* “*regulatory region variant*”; and 7) intergenic-not-regulatory SNPs those annotated as “*non coding transcript variant*”*,* “*upstream gene variant*”*,*“*downstream gene variant*”*,* “*feature truncation*”*,* “*intergenic variant*”. The results of the SNP annotation enrichment analysis are presented in the main text Figure 5 and in table S5.

To further test for enrichment of putatively regulatory sites among targets of LTBS we also used RegulomeDB, a SNP-based annotation for known and predicted regulatory elements (Boyle et al. 2012). We considered both each RegulomeDB score separately as well as a combination of score: 1a+1b+1c+2a+2b+2c, 1d+1e+3a+3b, and 1f+5, as these represent SNPs with the highest evidence for regulatory function (Boyle et al. 2012). As for other enrichment analyses, we considered the sum of scores across candidate windows, and obtained the expectation in the absence of LTBS by randomly re-sampling background windows (as above), generating a distribution used to assign empirical *p*-values for the enrichment of these scores. Results are provided in table S5.

**Supplementary Information S5. Candidate genes found in previous scans**

Many of the genes identified have previous evidence of balancing selection. We considered 258 genes reported in 3 scans for balancing selection (Andrés et al. 2009; Leffler et al. 2013; DeGiorgio et al. 2014). We identify three of the six genes with strong evidence of trans-species polymorphisms identified in (Leffler et al. 2013) (*HUS1*, *IGFBP7* and *PROKR2*). We also identify the five genes identified by both an exon-based approach (Andrés et al. 2009) and a genome-wide approach (DeGiorgio et al. 2014) (*HLA-B*, *CDSN, LGALS8, SLC2A9, RCBTB1*)*.* Overall, 89 (of 265) of the outlier genes, and 175 (of 1,594) of significant genes are found in previous scans. We also identify signatures of LTBS in the blood-group related *ABO* locus (*p* < 0.0008 in all populations), a well-known case of LTBS in humans (Ségurel et al. 2012; Ségurel et al. 2013). Because it is annotated by Gencode as a ‘processed transcript’ (version 19), it is not included in our gene tables*.*

We find45%of the genes from (Andrés et al. 2009) among the outliers (and 78% among the significant) and 10% and 38% of genes from(DeGiorgio et al. 2014) among outlier and significant genes, respectively.

**Supplementary Information S6. Increased heterozygosity in archaic genomes for regions detected as being under balancing selection in modern humans.**

***Method***

We used the high coverage genomes for three archaic individuals, Altai, Vindija and Denisova (Green et al. 2010; Meyer et al. 2012; Prüfer et al. 2013). We used assemblies generated with quality thresholds of mapping quality of at least 25 and 100mer mappability. For each tested *NCD* window we counted the number of callable bp (mappable to hg19), and the number of heterozygous positions for each of the archaic genomes. To be considered in this analysis, a *NCD* window needed to have at least 1500 bp (i.e. at least 50%) callable in the archaic genome. For each of the archaic genomes we calculated the heterozygosity for *NCD* candidate windows (both ‘significant’ and ‘outlier’) in each modern human population (gbr, tsi, yri, and lwk). Here heterozygosity is the number of heterozygous positions divided by the number of callable positions. Both the numerator and denominator are summed across all windows in each analysed window set. For comparison this same metric was calculated for 1,000 random samples (of size n = number of candidate windows) of analyzed windows,.

***Results***

For each of the archaic hominins we find significant increases in heterozygosity in regions we identify as showing signatures of balancing selection, when compared with the observed heterozygosity in non-candidate windows (all *p-values* < 0.001). This increase is greater for the more restrictive set of *outlier* windows (~ 15 times on average) than the *significant* ones (~ 4 times on average). For example, for candidates identified in GBR outlier windows contain 15.2 heterozygous positons per kb and significant windows 3.9, while the genome-wide background heterozygous rate is 0.2 (figure S22).

**Supplementary Figures**

**Fig. S1. *NCD* analytical properties as a function of number of SNPs.** *NCD2(0.5)* value (*y*-axis) by number of SNPs (*x*-axis). Colors correspond to a fixed FD value (20, 40, 100). *Top*, above ~1,500 SNPs (for any FD value), *NCD2(0.5)* stabilizes and asymptotically approaches 0. *Bottom*, a zoom-in. All SNPs have 0.5 frequency.

**Fig. S2. Analytical properties of *NCD2(0.5)*** **as a function of FD*.*** *NCD2(0.5)* value (*y*-axis) by number of FDs (*x*-axis). Colors correspond to different frequencies of the SNPs in the window. *Top*, when FDs ~500, *NCD2(0.5)* stabilizes and asymptotically approaches 0.5. *Bottom*, a zoom-in. All 20 SNPs have the same frequency. The minimum *NCD2(0.5)* value is different for the different colors (see information S1).

**Fig. S3. Effect of sequence length on *NCD2(0.5)*** **power (Africa).** ROC curves for sequence lengths 3 (left), 6 (middle) and 12 kb (right). Each plot shows *NCD2(0.5)* performance for simulations where the balanced polymorphism is modeled to achieve *f*eq = 0.5 (blue), *f*eq = 0.4 (orange), *f*eq = 0.3 (pink), based on simulations under the African demographic scenario and *Tbs* = 5 mya. FPR, false positive rate; TPR, true positive rate (sensitivity, or power).

**Fig. S4.** **Effect of sequence length on *NCD2(0.5)*** **power (Africa).** As in fig. S3, except *Tbs* = 3 mya.

**Fig. S5.** **Effect of sequence length on *NCD2(0.5)*** **power (Europe).** As in fig. S3.

**Fig. S6.** **Effect of sequence length on *NCD2(0.5)*** **power (Europe).** As in fig. S5, except *Tbs* = 3 mya.

**Fig. S7.** **Effect of sequence length on *NCD2(0.5)*** **power (Asia).** As in fig. S3.

**Fig. S8.** **Effect of sequence length on *NCD2(0.5)*** **power (Asia).** As in fig. S7, except *Tbs* = 3 mya.

**Fig. S9. Correlations between *NCD2(tf)* calculated with different *tf* values.** Each plot shows

the correlation between *NCD2* values calculated at two *tf* values. *NCD2* was calculated for

1,000 neutral simulations following demographic parameters for Africa. *L* = 3 kb.

**Fig. S10. ROC curves for comparison between *NCD2* and other tests.** Power to detect LTBS for simulations where the balanced polymorphism was modeled to achieve *f*eq = 0.3 (left), *f*eq = 0.4 (center), and *f*eq = 0.5 (left). Values are for European demography, *Tbs* = 5 mya, *L* = 3 kb. *tf* for *NCD* matches *f*eq. FPR, false positive rate; TPR, true positive rate. Power for other methods is shown in S1 Table.

**Fig. S11. Relationship between *NCD2(tf)*** **and the number of informative sites (IS)**

**based on simulations.** *NCD2tf* was calculated for neutral simulations (10,000 for each

IS value) for the African demographic scenario and the 0.01 quantile for each bin is

plotted. Blue (*tf* = 0.5), orange (*tf* = 0.4), pink (*tf* = 0.3), green (*tf* = 0.2). The dashed

line marks IS = 10. See also fig. S17.

**Fig. S12. Proportion of windows per chromosome.** Significant and outlier windows are derived from the union of three *tf* values for the LWK population. Windows: all scanned (grey), significant (green), outlier (blue).

**Fig. S13. Proportion of positions in the genome retained after each filter.** Proportion of the hg19 human reference genome (total base-pairs = 2,684,573,005) retained after application of each individual filter, and for all filters combined. Map50 = 0.843; TRF = 0.976; SD = 0.961; pantro2 = 0.961; Nr.IS = 0.939; all = 0.799. Map50: mappability 50-mer; TRF: tandem repeats; SD: segmental duplications; pantro2: reference chimp genome; Nr.IS, excluding windows with less than 10 IS (figs. S11 and S17) and/or < 500 bp of positions with orthology to chimp (see Methods in main text).


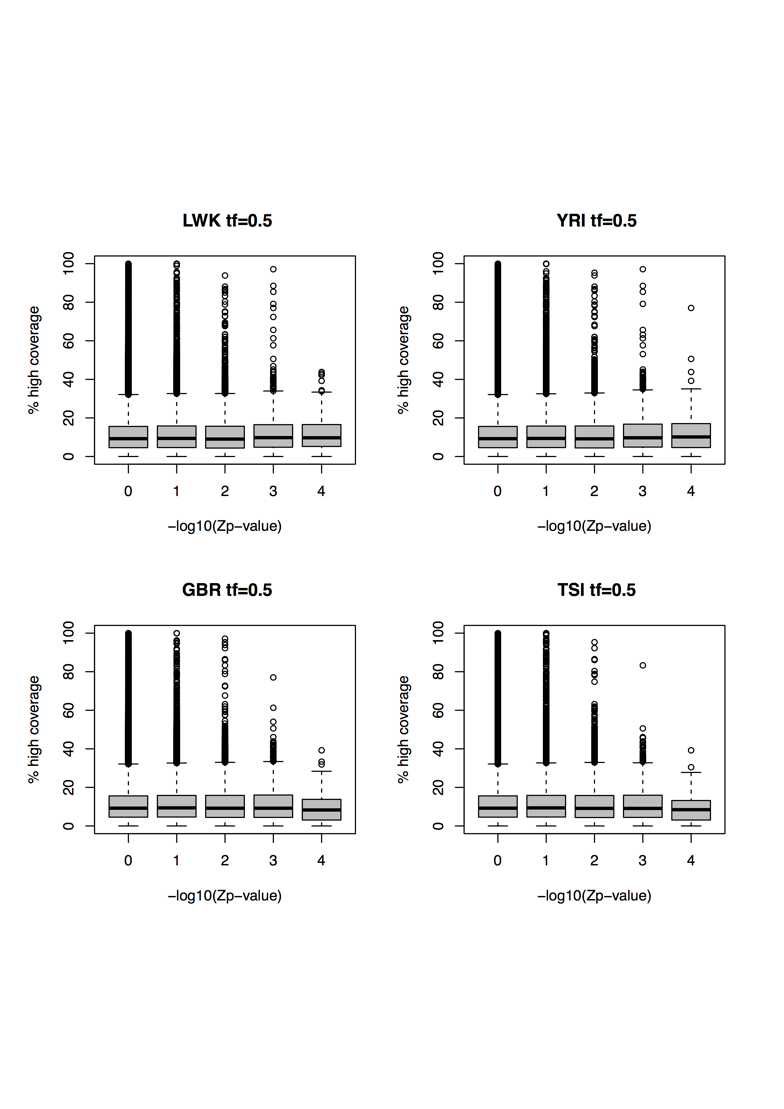
**
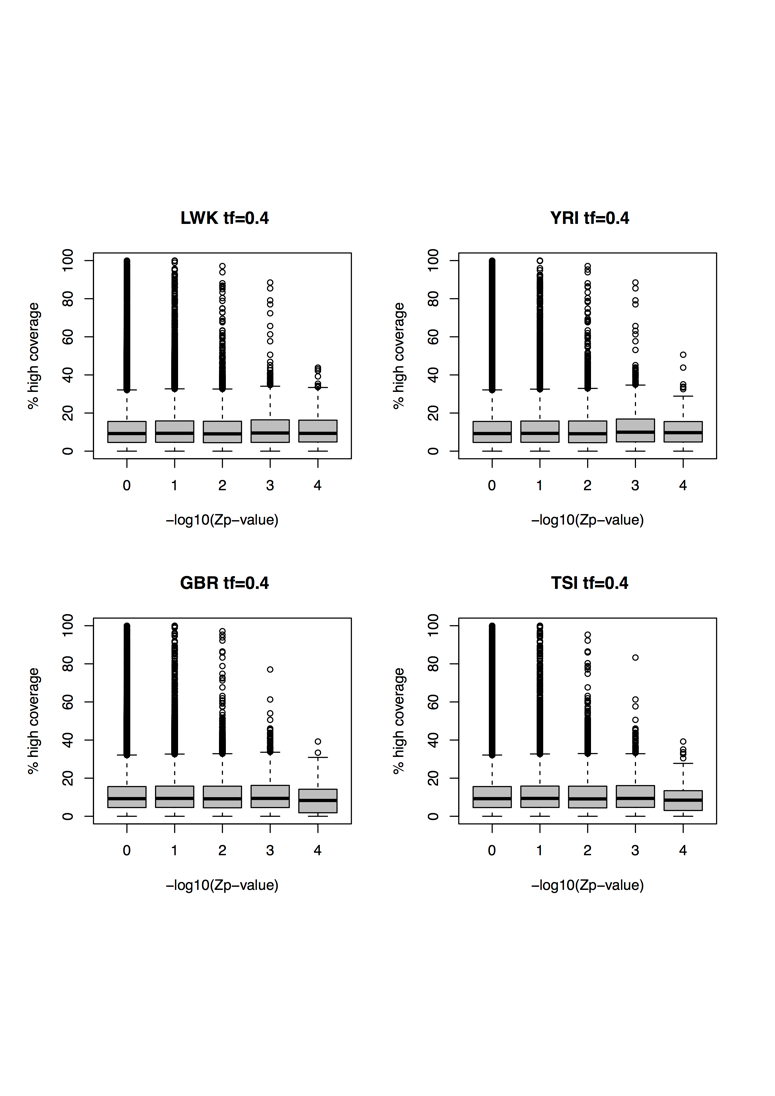

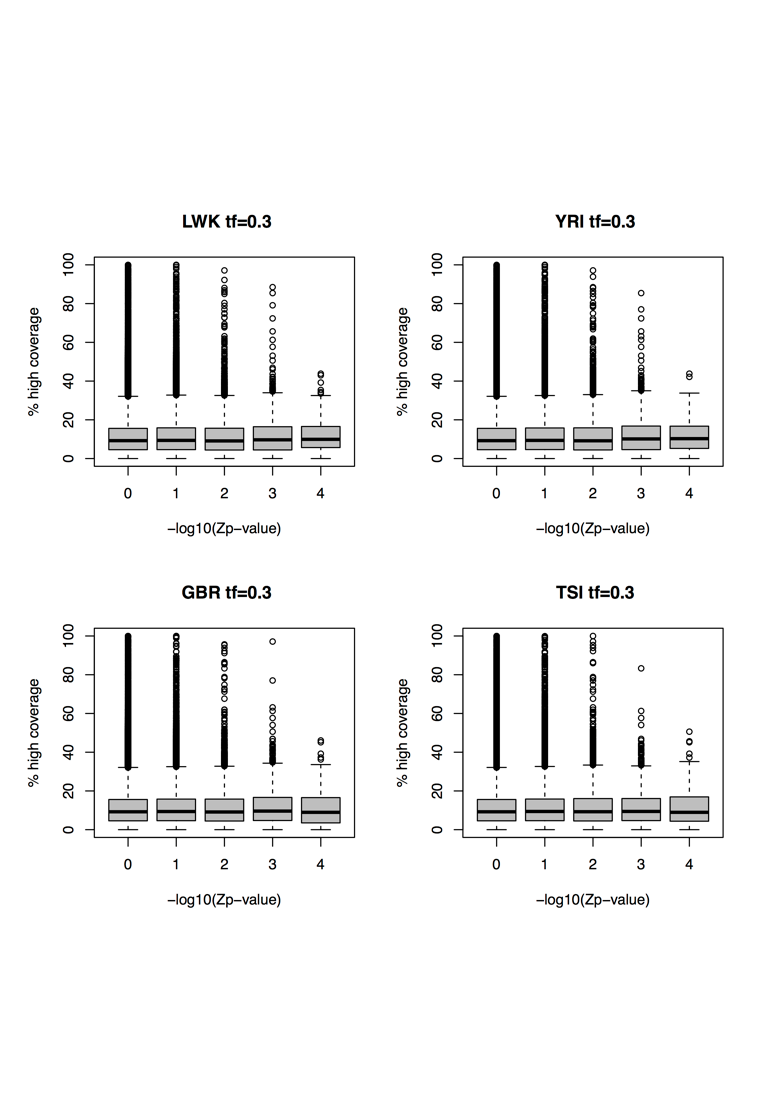
**

**Fig. S14. Distribution of proportion of high coverage positions per bin of empirical *NCD2* p-value.** *y*-axis, percentage of the sequence of a given window (of 3 kb) in this study having coverage values higher than the 97.5% quantile of the distribution in at least two samples of modern human shotgun data. *y*-axis values are binned by the *NCD*2 *Ztf-IS* empirical *p*-values (*Z*p-value) represented in –log10 scale on the *x*-axis. Results for all *tf*.

**Fig. S15. Number of paralogs on the same chromosome, per gene.** Proportion of genes (*y*-axis)

with a given number of paralogs on the same chromosome (*x*-axis). Only genes with at least

one annotated paralog (anywhere) were considered. “Genomic” refers to the scanned genes,

minus the significant genes from LWK (12,716 in total). “Significant_no_ORs” refers to the

union (all *tf*) of significant genes for LWK, minus those that are olfactory receptors (1,129

and 25 in total). Note. – more than 77% of significant and genomic genes have 0 or 1 paralog

in the same chromosome, compared to 12% of OR genes. Only 0.03% of the genomic genes

have more than 40 paralogs on the same chromosome, and none in the other sets, so these

values are not shown.

**Fig. S16. Proportion of putative Neandertal SNPs in the candidate windows of European populations.** In gray, distribution obtained from 1,000 re-samplings from background windows. In orange, % of Neandertal SNPs within all significant (or outlier) windows. TSI, Toscani; GBR, Great Britain. Results based on three introgression maps (information S3).

**Fig. S17. *NCD2(0.5)*** **empirical values and number of informative sites(violin plots).** *NCD2(0.5)* for windows with ISbetween 1-100 for (A) LWK and (B) GBR (>99% of all scanned windows have ≤ 100 IS). In blue, median value for all windows within a given bin. Orange dashed lines marks IS = 10, after which *NCD2* stabilizes.

**Fig. S18. Venn diagrams of candidate windows for four populations.** A, left, significant windows; B, right, outlier windows; YRI, Yoruba; LWK, Luhya (in tones of purple); GBR, Great Britain; TSI, Toscani (in tones of green). The sets of significant windows come from the union of candidate windows for all *tf* values (table 2 in main text).

**Fig. S19. Venn diagrams of significant windows for four populations, for each *tf* value.** From left to right:*tf* = 0.3; *tf* = 0.4, *tf* = 0.5. Populations names and colors as in fig. S18.

**Fig. S20. Venn diagrams of outlier windows for four populations, for each *tf* value.** As in fig. S19.

**Fig. S21. Venn diagrams of outlier genes for four populations, for each *tf* value.** As in fig. S20, but for protein-coding genes.

**S22 Figure. Proportion of heterozygous sites per mappable base pair between four human populations and three high coverage archaic genomes. See supplementary Information S6 for details.**

**Supplementary Tables**

**Table S1. Power analyses based on simulations.** Power to detect LTBS for simulation following African, European, and Asian demographic scenarios. False positive rate for reported power values is 5%. For ß we analyzed *Tbs* = 3 and *Tbs* = 5, which are the timescales we focus on the paper. For *T*1 and *T*2 we did not run all conditions because it is computationally expensive.

See attached spreadsheet.

**Table S2. Gene ontology and tissue-specific expression enrichment analyses**. For both types of analyses, four sets are shown for each population (LWK, YRI, GBR, TSI):1): significant (with and without HLA genes), outlier (with and without HLA genes). FDR, false discovery rate (only FDR < 0.20 cases are shown). *tf,* target frequency. Analysis performed with GOWINDA.

See attached spreadsheet.

**Table S3. Assigned *tf* values.** Candidate windows for all *tf*. Assigned *tf* are those that minimize the *p* - value for a given window (information S2). Percentage of windows with a given assigned *tf* in relation to the total is provided.

|  | **Pop/min tf** | ***tf* = 0.3** | ***tf* = 0.4** | ***tf* = 0.5** | ***tf* = 0.4 or *tf* = 0.3** | ***tf* = 0.5 or *tf* = 0.4** | **Total** |
| --- | --- | --- | --- | --- | --- | --- | --- |
|  |  |  |  |  |  |  |  |
| Significant | LWK | 1909(51%) | 770(21%) | 1027(28%) | 3(0%) | 6(0%) | 3715 |
|  | YRI | 2128(51%) | 933(22%) | 1084(26%) | 6(0%) | 5(0%) | 4156 |
|  | GBR | 1947(57%) | 745(22%) | 729(21%) | 2(0%) | 3(0%) | 3426 |
|  | TSI | 1841(54%) | 815(24%) | 737(22%) | 7(0%) | 5(0%) | 3405 |
|  |  |  |  |  |  |  |  |
|  |  |  |  |  |  |  |  |
|  | **Pop** | ***tf* = 0.3** | ***tf* = 0.4** | ***tf* = 0.5** | ***tf* = 0.4 or *tf* = 0.3** | ***tf* = 0.5 or *tf*= 0.4** | **Total** |
|  |  |  |  |  |  |  |  |
| Outlier | LWK | 217(52%) | 116(28%) | 83(20%) | 1(0%) | 4(1%) | 421 |
|  | YRI | 244(55%) | 112(25%) | 80(18%) | 5(1%) | 4(1%) | 445 |
|  | GBR | 234(56%) | 132(32%) | 49(12%) | 1(0%) | 1(0%) | 417 |
|  | TSI | 193(48%) | 156(39%) | 44(11%) | 5(1%) | 5(1%) | 403 |

Note.— Windows included here are those that are outlier or significant for all tf values. Pop, population. Min *tf*, target frequency that minimizes NCD2 for windows that are outlier or significant for all tf values.

**Table S4. Candidate genes and possibly introgressed NCD candidates.**

Protein-coding genes overlapped by candidate windows (union of all *tf*) in the two populations from each continent (Africa, YRI and LWK; Europe, TSI and GBR). African genes are ranked by: 1) *p* - value for LWK; 2) *p* - value for TSI; European genes are ranked by: 1) *p* - value for GBR; 2) *p* - value for TSI. “shared” genes are ranked by *p* - values in: LWK, YRI, GBR, TSI. The reported *p* - value is always the lowest one among all windows overlapping a given gene and the *tf* values is the one that minimizes the *p* - value (information S2). Possibly introgressed candidate genes: European candidate windows not shared with any African population, with proportion of introgressed SNPs ≥ 0.25 and the median MAF of introgressed SNPs ≥ 0.2. Reported genes are overlapped by candidate (outlier or significant windows) and by a Neandertal haplotype. See information S3.

See attached spreadsheet.

**Table S5. Bedfiles and SNP enrichment analyses.** Bedfiles: These correspond to the four populations (LWK, YRI, GBR, TSI), three target frequencies (0.3, 0.4, 0.5) and two sets (significant and outliers) of windows. Columns correspond to: chromosome, beginning of window, end of window. Coordinates based on hg19.Windows are ordered by position. Enrichment analyses: *p*-values obtained from 1,000 re-samplings from background windows, and for a one-tailed test where the alternative hypothesis is of enrichment in the candidate windows. Intermediate frequencies, only SNPs with MAF ≥ 0.20 are considered.

See attached spreadsheet.

**References**

Allison AC, Clyde DF. 1961. Malaria in African Children with Deficient Erythrocyte Glucose-6-phosphate Dehydrogenase. Br. Med. J. [Internet] 1:1346–1349. Available from: http://www.bmj.com/cgi/doi/10.1136/bmj.1.5236.1346

Andrés AM, Hubisz MJ, Indap A, Torgerson DG, Degenhardt JD, Boyko AR, Gutenkunst RN, White TJ, Green ED, Bustamante CD, et al. 2009. Targets of balancing selection in the human genome. Mol. Biol. Evol. [Internet] 26:2755–2764. Available from: http://www.pubmedcentral.nih.gov/articlerender.fcgi?artid=2782326&tool=pmcentrez&rendertype=abstract

Boyle AP, Hong EL, Hariharan M, Cheng Y, Schaub MA, Kasowski M, Karczewski KJ, Park J, Hitz BC, Weng S, et al. 2012. Annotation of functional variation in personal genomes using RegulomeDB. Genome Res. [Internet] 22:1790–1797. Available from: http://genome.cshlp.org/cgi/doi/10.1101/gr.137323.112

Charlesworth B, Charlesworth D. 2010. Elements of Evolutionary Genetics. 1st ed. Roberts and Company Publishers

DeGiorgio M, Lohmueller KE, Nielsen R. 2014. A model-based approach for identifying signatures of ancient balancing selection in genetic data. PLoS Genet. [Internet] 10:e1004561. Available from: http://www.pubmedcentral.nih.gov/articlerender.fcgi?artid=4140648&tool=pmcentrez&rendertype=abstract

Ewing G, Hermisson J. 2010. MSMS: a coalescent simulation program including recombination, demographic structure and selection at a single locus. Bioinformatics [Internet] 26:2064–2065. Available from: http://bioinformatics.oxfordjournals.org/cgi/doi/10.1093/bioinformatics/btq322

Gravel S, Henn BM, Gutenkunst RN, Indap AR, Marth GT, Clark AG, Yu F, Gibbs RA, Bustamante CD. 2011. Demographic history and rare allele sharing among human populations. Proc. Natl. Acad. Sci. U. S. A. [Internet] 108:11983–11988. Available from: http://www.pubmedcentral.nih.gov/articlerender.fcgi?artid=3142009&tool=pmcentrez&rendertype=abstract

Green RE, Krause J, Briggs AW, Maricic T, Stenzel U, Kircher M, Patterson N, Li H, Zhai W, Fritz MH-Y, et al. 2010. A draft sequence of the Neandertal genome. Science (80-. ). [Internet] 328:710–722. Available from: http://www.ncbi.nlm.nih.gov/pubmed/20448178

Gutenkunst RN, Hernandez RD, Williamson SH, Bustamante CD. 2009. Inferring the Joint Demographic History of Multiple Populations from Multidimensional SNP Frequency Data.McVean G, editor. PLoS Genet. [Internet] 5:e1000695. Available from: http://dx.plos.org/10.1371/journal.pgen.1000695

Hedrick PW. 2012. What is the evidence for heterozygote advantage selection? Trends Ecol. Evol. Evol. 27:698–704.

Leffler EM, Pfeifer S, Auton A, Venn O, Bowden R, Bontrop R, Wall JD, Sella G, Donnelly P, Mcvean G, et al. 2013. Multiple Instances of Ancient Balancing Selection Shared Between Humans and Chimpanzees. Science (80-. ). 339:1578–1582.

Liu X, Fu Y, Liu Z, Lin B, Xie Y, Liu Y, Xu Y, Lin J, Fan X, Dong M, et al. 2006. An Ancient Balanced Polymorphism in a Regulatory Region of Human Major Histocompatibility Complex Is Retained in Chinese Minorities but Lost Worldwide. Am. J. Hum. Genet. [Internet] 78:393–400. Available from: http://linkinghub.elsevier.com/retrieve/pii/S0002929707623799

Meyer D, Single RM, Mack SJ, Erlich HA, Thomson G. 2006. Signatures of demographic history and natural selection in the human major histocompatibility complex Loci. Genetics [Internet] 173:2121–2142. Available from: http://www.pubmedcentral.nih.gov/articlerender.fcgi?artid=1569707&tool=pmcentrez&rendertype=abstract

Meyer M, Kircher M, Gansauge M-T, Li H, Racimo F, Mallick S, Schraiber JG, Jay F, Prufer K, de Filippo C, et al. 2012. A High-Coverage Genome Sequence from an Archaic Denisovan Individual. Science (80-. ). [Internet] 338:222–226. Available from: http://www.sciencemag.org/cgi/doi/10.1126/science.1224344

Prüfer K, Racimo F, Patterson N, Jay F, Sankararaman S, Sawyer S, Heinze A, Renaud G, Sudmant PH, de Filippo C, et al. 2013. The complete genome sequence of a Neanderthal from the Altai Mountains. Nature [Internet] 505:43–49. Available from: http://www.nature.com/doifinder/10.1038/nature12886

Sanchez-Mazas A. 2007. An apportionment of human HLA diversity. Tissue Antigens [Internet] 69:198–202. Available from: http://doi.wiley.com/10.1111/j.1399-0039.2006.00802.x

Sankararaman S, Mallick S, Dannemann M, Prüfer K, Kelso J, Pääbo S, Patterson N, Reich D. 2014. The genomic landscape of Neanderthal ancestry in present-day humans. Nature [Internet] 507:354–357. Available from: http://www.ncbi.nlm.nih.gov/pubmed/24476815

Ségurel L, Gao Z, Przeworski M. 2013. Ancestry runs deeper than blood: The evolutionary history of ABO points to cryptic variation of functional importance. BioEssays [Internet] 35:862–867. Available from: http://doi.wiley.com/10.1002/bies.201300030

Ségurel L, Thompson EE, Flutre T, Lovstad J, Venkat A, Margulis SW, Moyse J, Ross S, Gamble K, Sella G, et al. 2012. The ABO blood group is a trans-species polymorphism in primates. Proc. Natl. Acad. Sci. [Internet] 109:18493–18498. Available from: http://www.pnas.org/cgi/doi/10.1073/pnas.1210603109

Solberg OD, Mack SJ, Lancaster AK, Single RM, Tsai Y, Sanchez-Mazas A, Thomson G. 2008. Balancing selection and heterogeneity across the classical human leukocyte antigen loci: A meta-analytic review of 497 population studies. Hum. Immunol. [Internet] 69:443–464. Available from: http://linkinghub.elsevier.com/retrieve/pii/S0198885908000803

Tan Z, Shon AM, Ober C. 2005. Evidence of balancing selection at the HLA-G promoter region. Hum. Mol. Genet. [Internet] 14:3619–3628. Available from: http://www.hmg.oxfordjournals.org/cgi/doi/10.1093/hmg/ddi389

Teixeira JC, de Filippo C, Weihmann A, Meneu JR, Racimo F, Dannemann M, Nickel B, Fischer A, Halbwax M, Andre C, et al. 2015. Long-Term Balancing Selection in LAD1 Maintains a Missense Trans-Species Polymorphism in Humans, Chimpanzees, and Bonobos. Mol. Biol. Evol. [Internet] 32:1186–1196. Available from: http://mbe.oxfordjournals.org/cgi/doi/10.1093/molbev/msv007

Vernot B, Akey JM. 2014. Resurrecting Surviving Neandertal Lineages from Modern Human Genomes. Science (80-. ). [Internet] 343:1017–1021. Available from: http://www.sciencemag.org/cgi/doi/10.1126/science.1245938

Vernot B, Tucci S, Kelso J, Schraiber JG, Wolf AB, Gittelman RM, Dannemann M, Grote S, Mccoy RC, Norton H, et al. 2016. Excavating Neandertal and Denisovan DNA from the genomes of Melanesian individuals. Science (80-. ). 383:235–239.
